# Supplementary material for: ToxPi*GIS Toolkit: Creating, viewing, and sharing integrative visualizations for geospatial data using ArcGIS
Source: medRxiv. 2021 Oct 12:2021.10.08.21264756. Preprint. [Version 2] doi: 10.1101/2021.10.08.21264756 (PMC8528084; doi:10.1101/2021.10.08.21264756)
Supplement: 1 [file NIHPP2021.10.08.21264756V2-supplement-1.pdf]

medRxiv preprint doi: https://doi.org/10.1101/2021.10.08.21264756; this version posted October 12, 2021. The copyright holder for this preprint (which was not certified by peer review) is the author/funder, who has granted medRxiv a license to display the preprint in perpetuity. This article is a US Government work. It is not subject to copyright under 17 USC 105 and is also made available for use under a CC0 license.

SUPPLEMENTAL and OTHER OPTIONAL MATERIAL

All applications, usage instructions, sample data, example visualizations, and open-source code are freely available from a dedicated GitHub page linked from www.toxpi.org. Direct links to specific elements described above are provided here.

ToxPi*GIS Toolkit Github

https://github.com/Jonathon-Fleming/ToxPi-GIS

Vignette1: *ToxPi_creation.py* Demonstration

https://ncsu.maps.arcgis.com/home/item.html?id=7c0365b3f75949369b46c07ae4ecf10c

Vignette2: *ToxPi_creation_customized.py* Demonstration

https://ncsu.maps.arcgis.com/home/item.html?id=1518637a0b454036a3d0d2fc8239ff08

HotSpot/Dashboard Demonstration

https://ncsu.maps.arcgis.com/home/item.html?id=022416cbc74d430691ad7d2a4cbec229
